# Disgust-reduction evaluative conditioning (DREC) and brain stimulation in patients with contamination-based obsessive-compulsive disorder: a protocol for a randomized control trial

**DOI:** 10.1186/s13063-023-07791-2

**Published:** 2023-11-24

**Authors:** Faezeh Raeis Al Mohaddesin, Ali Moghimi, Javad Salehi Fadardi

**Affiliations:** 1 Department of Biology, Faculty of Science, Ferdowsi University of Mashhad, Mashhad, Iran; 2 Rayan Research Center for Neuroscience and Behavior, Department of Biology, Faculty of Science, Ferdowsi University of Mashhad, Mashhad, Iran; 3 School of Community and Global Health, Claremont Graduate University, Claremont, USA; 4 Department of Psychology, Faculty of Education and Psychology, Ferdowsi University of Mashhad, Mashhad, Iran

**Keywords:** Obsessive-compulsive disorder, Contamination symptom, Disgust, Evaluative Conditioning, Transcranial direct current stimulation, Orbitofrontal cortex

## Abstract

**Background:**

The negative emotional valence of a stimulus can be altered if paired with a pleasant stimulus, a phenomenon referred to as evaluative conditioning. Disgust, as a central emotion in obsessive-compulsive disorder (OCD), particularly in the contamination subtype, may be an appropriate target for such a method. We know that disgust processing and OCD pathophysiology share in some brain areas, including the orbitofrontal cortex, as the neuromodulation techniques targeted in this area have been able to decrease OCD symptoms. We aim to conduct a randomized clinical trial to investigate the evaluative conditioning effect on disgust reduction in patients with contamination-based OCD when administered with or without neuromodulation targeted orbitofrontal cortex.

**Method:**

In a single-blind randomized control trial (RCT), 55 patients with contamination-based OCD will be randomly assigned to four arms. In a factorial design, they will receive 10 sessions of evaluative conditioning training (either sham or real) plus cathodal transcranial direct current stimulation (tDCS) over the orbitofrontal cortex (either sham or real). The intensity of disgust experience and clinical symptoms will be investigated as primary outcomes and quantitative electroencephalogram and cognitive functions as secondary outcomes. The data will be collected at three assessment levels: baseline, after completing intervention sessions, and 2-month follow-up.

**Discussion:**

The present RCT is the first study that applies evaluative conditioning training in the OCD clinical sample. It will clarify the effect of the evaluative conditioning method alone and with tDCS on disgust reduction in patients with contamination-based OCD. It will provide initial evidence for such an emotion modulation method in the OCD population. The effect of this emotion-focused protocol on cognitive functions and electroencephalogram components is also of interest.

**Trial registration:**

ClinicalTrials.gov, NCT05907369. Registered on 16 June 2023. Retrospectively registered.

**Supplementary Information:**

The online version contains supplementary material available at 10.1186/s13063-023-07791-2.

## Background

Obsessive-compulsive disorder (OCD) is a disabling mental with a lifetime prevalence of 2–3% [1, 2]. It is known by repetitive, unwanted, persistent thoughts, images, or urges as obsessions and repetitive behaviors or mental rituals as compulsions. It reduces the quality of life since it is accompanied by cognitive, social, and occupational deficits [3, 4]. Considering the symptom diversity, nearly half of OCD patients are concerned about dirt, germs, affections, and diseases, accompanied mainly by excessive washing and cleaning behaviors [5], called contamination-based OCD (C-OCD). It has become more critical since a considerable increase in C-OCD prevalence has occurred in recent years due to the COVID-19 pandemic [6, 7].

It is believed that disgust is an essential emotional characteristic of OCD and has a central role in the development, maintenance, and treatment of the disorder [8], particularly in C-OCD, so Curtis [9] claimed that “the disorder of disgust system” is a more appropriate description for this mental condition. Disgust showed a stronger correlation with contamination symptoms [10, 11]. Supposed that the functional role of disgust is to keep us away from dirt and diseases, C-OCD seems to be an exaggerated disgust processing, including a false contamination alarm that resulted in excessive avoidant behavior. The C-OCD sufferers experience abnormal mental contamination, meaning they feel contaminated despite no physical contact with an actual contaminant [12]. Dysfunctional disgust processing is the culprit for such a false contamination alarm [8].

There are a growing number of studies that targeted disgust in OCD. For example, Fink et al. [13] reduced disgust in C-OCD patients through two emotion-regulation techniques: imagery rescripting and cognitive reappraisal. Other methods are also administered to reduce disgust, such as exposure-based cognitive-behavioral therapy [14], imagery rescripting combined with brain stimulation [15], and virtual reality (VR) exposure [16]. Disgust reduction corresponds with improvements in C-OCD symptoms [14, 17], and disgust proneness (i.e., how much one is prone to experience disgust) is associated with treatment results [18, 19]. It also showed an association with treatment results at the follow-up level [20].

In the recent book on disgust, Reynolds and Askew concluded that disgust is mainly developed through evaluative conditioning (EC) [21]. EC is a process through which pairing a neutral-valence stimulus (conditioned stimulus; CS) with an emotional-valence stimulus (unconditioned stimulus; US) could result in a change in the emotional valence of the CS. So, it can change the amount of liking or disliking of the target stimulus (i.e., CS). The change is referred to as the “EC effect.” EC is resistant to extinction [22] because extinction does not target the valence of CS. Therefore, extinction may not appropriately reduce disgust emotion [23].

Although exposure-response prevention (ERP) is the first-line behavioral treatment for OCD, there are inconsistencies in the effectiveness of exposure-only to reducing disgust [20, 24]. The exposure-only technique seems successful in reducing fear rather than disgust. It may be due to the role of Pavlovian conditioning in producing fear responses and its sensitivity to the extinction phenomenon, compared with the role of evaluative conditioning in disgust reactions and its resistance to extinction [25]. Disgust resistance to extinction is an important obstacle toward treatment, particularly for those with higher disgust proneness [23]. C-OCD patients, compared with other OCD subtypes, experience higher levels of disgust and move more slowly through the habituation process in the ERP interventions [26].

Another method known as counterconditioning may be effective in modifying CSs with disgust valence. In counterconditioning, a CS is paired with a US with opposite valence. Pairing disgusting stimuli with pleasant USs seems to be a preferred treatment approach for reducing disgust even in OCD patients [24, 25]. Evaluative counterconditioning has led to a decrease in disgust valence in OCD analog samples with contamination concern [27] and in other disgust-relevant conditions like body dissatisfaction [28]. Considering the central role of disgust in C-OCD, EC may have therapeutic implications for clinical OCD populations, but the effectiveness for the OCD population has remained unclear.

OCD pathophysiology shares neurocircuits and brain structures involved in disgust processing [29]. Among them are the insular cortex and orbitofrontal cortex (OFC). Insular activity is a neural correlate for disgust processing [30, 31]. Insula shows regular higher activity in experiencing actual disgusting stimuli, but the abnormality is when an OCD patient confronts a symptom-provoking stimulus that is not genuinely disgusting. In this situation, insular hyperactivity showed that non-disgusting stimuli are mistakenly perceived as highly disgusting [32]. The insula sends some projections to the OFC [33, 34], the area engaged in coding the aversive value of the stimuli [35], and its enhanced activity, among the insula and other areas, is associated with avoidance behavior in a disgust-relevant situation [36]. The OFC also showed increased gray matter volume in people with higher disgust sensitivity (how much they are distressed when experiencing disgust) [37]. Neuroimaging studies reported the overactivation of OFC in OCD patients [38]. The neuromodulation techniques in this area, such as transcranial direct current stimulation (tDCS), have a therapeutic effect on OCD symptoms [39, 40].

The present study aims to design an EC training targeting disgust toward contamination-related stimuli in C-OCD patients. The hypothesis is that pairing such stimuli with pleasant USs may decline the disgust evoked. The proposed intervention will target lings of disgust more directly and precisely. We will also investigate the effect of brain stimulation over the OFC on disgust processing in C-OCD people. Finally, we are interested in whether adding this neuromodulation technique to the EC training may boost the EC effect. So, we will compare the effectiveness of individual and combined interventions in reducing disgust reactivity.

We hypothesize that EC and brain stimulation would decrease disgust experience when exposed to contamination-related stimuli (primary outcome), which, in turn, would improve OCD symptoms (primary outcome). Moreover, since emotional processing can interfere with cognitive functions [41], it is hypothesized that decreases in disgust reactivity would be associated with increases in cognitive functions, particularly in one’s disgust-related attentional bias and inhibitory control (secondary outcomes). We will use electroencephalographic (EEG) characteristics to examine the effectiveness of EC, brain stimulation, and their combined use combination of the (secondary outcome).

Below is a detailed description of the proposed randomized control trial (RCT) following the SPIRIT framework [42].

## Methods/design

### Study design and setting

**Fig. 1:**
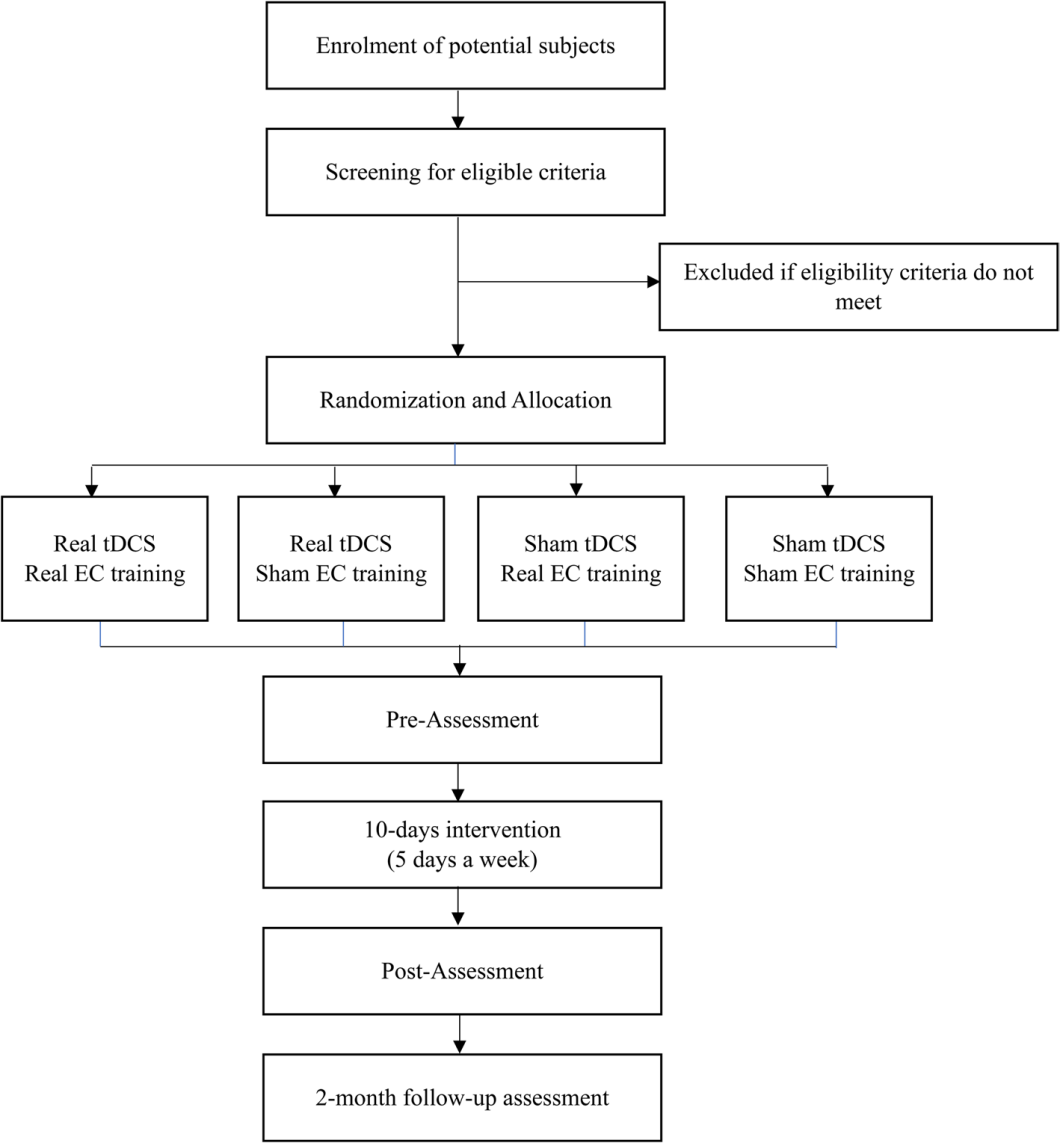
CONSORT flow chart

This study is a four-arm, randomized, placebo-controlled clinical trial in which participants get sham or real forms of two interventions, including EC and tDCS. It is aimed to investigate the effectiveness of EC, tDCS, and a combination of both on disgust reduction, clinical symptoms, and cognitive characteristics in C-OCD patients. Randomly assignment will be in blocks of 4 in 1:1:1:1 allocation to four parallel groups: (1) real EC plus real tDCS, (2) sham EC plus real tDCS, (3) real EC plus sham tDCS, (4) sham EC plus sham tDCS. We consider sham interventions to be able to separate the exposure effect from the EC effect and also to monitor the placebo effect. Assessments occur at baseline, after completion of 10 interventional sessions, and in a 2-month follow-up. All groups have similar assessments and equal intervention amounts (equal time, activity, and number of sessions). Figure 1 shows the design of the study. The trial will be conducted in the Cognitive Science Laboratory of Ferdowsi University of Mashhad, Mashhad, Iran.

### Participants

Eligibility criteria include the following: aged 18–55 years; diagnosed with OCD based on The Diagnostic and Statistical Manual of Mental Disorders-Fifth Edition-Text Revision (DSM-V-TR) with significant contamination-based symptoms; sufficiently able to read, write, and do computerized tasks; being on stable medication for at least the last 3 months; and filling out the written consent.

The potential participants will be excluded if one or more of the following conditions are present: diagnosis of psychotic disorders or another comorbid with severe symptoms, alcoholism or drug abuse, a severe medical condition that may restrain the ability to engage in assessments and interventions, “Yes” answer to any items of screening questions for transcranial electric stimulation (tES) [43], invalid response on baseline tests.

Those participants who receive new medical prescriptions or other brain stimulations during the administering trial or follow-up durations will be excluded from further assessments or analysis.

### Recruitment

Through a research agreement, several psychiatrists and psychotherapists will refer participants to the researchers. Study ads and flyers will be available in clinics for C-OCD patients as potential subjects. Self-referral will be another path. They will also be recruited via online advertising on social media as well as the distribution of the study ads and flyers among university students. Online advertisements and flyers provide some information about the trial and inclusion criteria. Interested candidates will be invited to an initial meeting and baseline assessment within 3 days of enrollment. Candidates informed by flyers or advertisements will also pass a diagnosis interview by a therapist at the first session to check full inclusion criteria.

### Material

The computerized tasks and training will be pictorial. The pictures should belong to one of three classes: (1) contamination-related pictures that elicit disgust, (2) pleasant pictures that elicit positive emotions such as joy or calmness, and (3) neutral pictures that elicit no particular emotion. We have collected a picture series from internet websites containing 30 pictures in each class. In collecting procedures, we dynamically screened pictures based on the comments and ratings of people other than the research team (healthy and OCD people). Ultimately, a collection of 90 pictures was selected (30 for each class). In a final pilot rating, a group of 15 patients with C-OCD symptoms was required to rate them on a scale from − 10 (very disgusting) to 0 (neutral; not disgusting-not pleasant) to 10 (very pleasant) to ensure their emotional value and significant differences between classes.

#### Conditioned stimuli

In a survey asking people with OCD about contamination concern-related situations and stimuli that elicit disgust and emotion, a list of items was obtained. It included good examples of stimuli to be targeted as CSs. Based on the list, we have collected pictures containing those stimuli. The pictures visibly contained the contamination about OCD people are experiencing concern or obsessions. It might be an exaggeration of real-life situations for some pictures. For example, OCD people show obsessions, “What if the toilet is not completely clean,” even if any dirtiness is obvious, and we had to select a picture of a really dirty toilet to elicit that emotion. These include 30 colorful pictures (e.g., a dirty hand, a muddy face, a pile of unwashed clothes, a dirty sink) rated between − 7.5 to − 10 (mean score= − 9.44) and will be applied in developing an EC training as CSs. Some will also be applied for cognitive tests as emotionally disgusting stimuli.

#### Unconditioned stimuli

Unconditioned stimuli will consist of pleasant pictures (positive-valence unconditioned stimuli; pUS), used in real EC training, or completely neutral (neutral-valence unconditioned stimuli nUS), used in sham EC training. The pUSs will be those 30 pleasant pictures selected in the picture-collecting procedure and rated between 7.5 to 10 in the pilot rating (mean score = 9.36). They are colorful pictures such as a smiling face, a cute baby, the nature of winter, spring, the sooth waves of the sea, and beautiful flowers. All can elicit positive emotions and not seem to be related to any compulsions. In contrast, the nUSs will include geometrical figures (e.g., circle, rectangular) or symbols (e.g., percent, omega), all in white color set on a black background.

#### Neutral stimuli

We have also provided several colorful pictures that do not elicit any particular emotions. The neutral collection includes 30 pictures rated from − 1.5 to + 1.5 (mean score = 0.92) in the pilot rating. They will be the neutral pictorial stimuli in cognitive tasks.

### Interventions

The interventions will comprise EC training and brain stimulation via tDCS.

EC training is a kind of emotion-focused associative learning. The valence of a CS may alter via pairing it with an emotionally valanced US. Recently, it has been proposed as a strategy for modifying emotional components in normal or clinical populations [44]. Fear and disgust are the most negative emotions targeted for such a strategy [24, 45], in which the stimuli that elicit fear or disgust pair with the US of positive valence. This pairing may decrease their negative valence, so feared or disgusting stimuli are experienced as less unpleasant than before. Based on the literature that implicates EC as potentiation for targeting disgust in OCD [25], we will administer an innovative EC paradigm with the details below, combined with or without brain stimulation using tDCS.

tDCS is a non-invasive brain modulation method that may change cortical excitability by applying a weak direct current between two electrodes (anode and cathode) positioned on the scalp. An increase or decrease in excitability is due to anodal or cathodal tDCS [46]. The dorsolateral prefrontal cortex (DLPFC), pre-supplementary motor area (pre-SMA), and OFC constitute the major targets of electrode montage for OCD [47]. This RCT will target the OFC, one of the primary brain regions engaged in OCD and disgust [48].

Each intervention will have two forms: active (real) and inactive (sham).

#### Active intervention

##### Real EC training

The EC task has been inspired by Kosinski’s research [28], who developed a brief game-like mobile app targeting body dissatisfaction. We have designed a computerized version with contamination-related stimuli for ten therapeutic sessions. We have set it in four difficulty levels, nearly the same as Kosinski. Our EC task will include 30 CS-US pairs using 30 contamination-related pictures as CSs and 30 pleasant pictures as USs.

In sessions 1 to 3 (difficulty level 1), ten pairs will be presented in each session. The task will consist of 4 blocks. Each of the first three blocks will start with a pair list shown on the screen at a time (including 3 pairs for the first block, 3 for the second, and 4 for the third one, a total of 10 pairs of pictures). The list will remain for 30s in the first two blocks and 40s in the third. Participants will be asked to memorize all the picture pairs. The subsequent trials will provide one of the CSs on the top of the screen and three US options on the bottom. Participants will be instructed to select the appropriate US as quickly and accurately as possible. Each pair will repeat 18 times in the first three blocks. Before starting the last block, there will be a rest time for 30s. Finally, the last block, including 60 trials, will review all 10 picture pairs with six repetitions each. The answer options will be presented 300 ms after the CS presentation starts and remain on the screen until the correct option is selected.

In sessions 4 to 6 (difficulty level 2), the answer options will remain for 400 ms and then disappear. Therefore, the participants should be more concentrated, find the correct answer, and memorize its location to choose after disappearing from the blank picture frame. The other components will be the same as before.

In each session of 7 to 10, 15 pairs will be practiced, with five pairs presented on the pair list of the first three blocks remaining for only 7 s to be remembered, and the subsequent trials will consist of 12 repetitions of each. The last block will include 60 trials reviewing all 15 pairs. The answer options will increase to 5, presented 300 ms after the CS presentation starts. For sessions 7 and 8 (difficulty level 3), the options will remain on the screen until selecting the correct answer. However, the options for sessions 9 and 10 (difficulty level 4) will be presented only for 400 ms.

The inter-trial interval (ITI) will be 1000 ms for all levels. Participants will answer to 240 conditioning trials in each training session which will take about 15 min to complete.

##### Real tDCS

Stimulation will be provided using the standard device (Activadose DC stimulator) with two rectangular saline-soaked sponge electrodes placed based on the international 10-20 electrode placement system. The cathode (size 5 × 7 cm^2^) will be placed over the left OFC (FP1) and the anode (size 10 × 10 cm^2^) over the right cerebellum (3 cm below the inion and 1 cm right of the midline). This montage is similar to that of Bation et al. [39], but increasing the size of the reference electrode (anode) is due to have a more focal cathodal effect over the left OFC. The tDCS will be delivered with the current intensity of 2 mA for 20 min for 10 sessions (5 days a week).

#### Inactive interventions

##### Sham EC training

For sham EC training, all the details will be the same as active EC training except that we will use nUSs instead of pUSs.

##### Sham tDCS

With the same device and electrode montage as active tDCS, the sham condition will include a current as low as 0.1 mA to be sure that current density is below the minimum threshold of modulating cortical activity, i.e., 0.017 mA/cm^2^ [46].

### Procedure

The enrolled candidate will be invited to an initial interview to confirm eligibility. They also will be provided with detailed information about the trial and procedure and then fill out written consent. Participants who will not meet the eligibility criteria or will not interested anymore will be excluded from the trial. The interview will continue with qualified volunteers in order to record demographic information such as age, gender, education level, age of onset, history of OCD in the family, taking medication or not (if yes, what, since when, and stability duration), and other receiving treatment (past and present) if any.

Participants will be encouraged not to be sleepy or hungry at all sessions in order to be able to focus on tasks appropriately. They will be informed to avoid these conditions in addition to smoking and caffeine consumption from the night before EEG recording.

Once participation is confirmed, an OCD patient will be assigned to one of four trial conditions based on a randomized allocation. A baseline assessment will consist of brain wave recording, computerized cognitive tests, and an online version of self-report measures; it will take about 60 min to complete. After that, as shown in the CONSORT diagram (see Fig. 1), all participants will complete a 10-session intervention. Each session will start with brain stimulation (sham or real); after 5 min, they will be asked to do EC training simultaneously.

### Adverse effect monitoring

The stimulation protocol has been reported as safe and suitable for OCD symptom reduction in previous RCTs [39, 40], and there is no anticipated harm and compensation for trial participation. However, if a participant reports any moderate to serious problem, the stimulation will be stopped, and the trial will be discontinued for that participant, and if more than two participants report serious adverse events, the stimulation will be discontinued. According to the ethics committee rules, the trial management group is responsible for providing any necessary medical care due to trial participation.

### Measures

#### Screening measure for tDCS

Antal et al. [43] have provided a comprehensive measure to screen all candidates for tES. The contraindications and high-risk conditions for tES will be listed in a questionnaire, including a history of epilepsy or seizure, having metal or electronic implants in the head, use of a cardiac pacemaker, history of head trauma or head surgery, being pregnant, and history of fainting spells or syncope.

#### Pre-, post-, and follow-up assessments

##### Quantitative electroencephalography (qEEG)

EEG is a non-invasive technique that allows recording the brain’s electrical activity over the scalp. Extracting EEG signal features through mathematical algorithms is called quantitative EEG, an applicable method in research and clinical diagnosis for neurological and psychiatric disorders [49]. For the present study, EEG will be recorded by an EEG equipment amplifier using a 19-electrode cab placed according to the international 10-20 system. EEG recording will be obtained during two resting conditions, including eyes-closed and eyes-open, for at least 3 min for each condition. Participants will be required to avoid caffeine, tobacco, and alcohol from the night before recording. They will be provided with some explanations to be familiarized with the recording procedure before starting. After fitting into the cab, they are asked to sit comfortably and close their eyes for eyes-closed recording. Then, they open their eyes and fixate on the screen with minimum eye blinking for eyes-open recording. The quantitative analysis will be performed by referring recorded data to the database.

##### Cognitive tests

The computerized cognitive tests include the following:

- Dot-probe test (DPT) assesses attentional bias toward contamination-related stimuli. DPT trials consist of simultaneously presenting two stimuli on the screen. When they disappear, a probe is shown at the location of one of them. Reaction time to the probe change depends on the attentional bias toward one of two pictorial stimuli [50, 51].
- Emotional Stop-Signal test (ESST) to see to what extent the emotions interfere with inhibitory control. The standard SST is a choice reaction time task in which each of two target stimuli (e.g., a right and a left arrow) correspond with a key press response (e.g., right and left key). Participants should respond as quickly as possible by pressing the corresponding response key (go-trials) and withhold response if the stimulus is followed by a stop-signal (e.g., a tone; stop-trials). The ESST includes emotional stimuli presenting before targets with the assumption that emotion may disrupt inhibitory control [52].
- Emotional Go/NoGo test (EGNT) to investigate how emotions interfere with response inhibition. The participants are instructed to respond as quickly as possible to go-trials (e.g., a symbol $) and avoid responding to no-go-trials (e.g., a symbol #). By presenting emotion-eliciting stimuli (e.g., before the target stimuli), their interference effect on response inhibition can be explored [53, 54].

The stimuli will be selected from contamination-related pictures (the same CSs of training) that are disgust-eliciting and neutral pictures.

##### Self-report measures

The self-report measures include the following:

- Padua Inventory-Washington State University Revision (PI-WSUR), a 39-item scale to measure OCD symptoms in 5 subscales, rating on a 5-point Likert scale (0 = not at all, to 4 = very much). It includes ten items for the “contamination obsessions and washing compulsions” subscale [55, 56].
- Yale-Brown Obsessive-Compulsive Scale (Y-BOCS) that is one of the most applied scales to assess the severity of OCD symptoms; its ten items address the amount of time, interference, distress, resistance, and control for obsessions and compulsions separately. All are rated 0 to 4 [57].
- Disgust Scale-Revised (DS-R), a questionnaire comprising 25 items that describes common disgust-relevant experiences. The first part (items 1–13) requires answers to true/false choices and the second part (items 14–25) requires a rating on not/slightly/very choices. Core disgust, animal reminder disgust, and contamination-based disgust are the three subscales [58].
- Beck Depression Inventory-II (BDI-II), the revised version of BDI, consists of 21 items. All items reflect major depression disorder (MDD) symptoms based on DSM-IV and should be ranked on a 4-point scale [59].
- Beck Anxiety Inventory (BAI), a 21-item scale to rate anxiety symptoms severity on a scale ranging from 0 (not at all) to 3 (severely) over the last week [60].
- Disgust Rating Scale (DRS), a self-report evaluation in which the participants will rate all 30 CSs in terms of how much they can elicit disgust on a scale from 0 (not at all) to 10 (very much).

BDI-II, BAI, and DS-R scores will be administered once at pre-assessment and will not repeat at post- or follow-up assessments, but others do.

### Expected outcomes

This trial will investigate whether positive EC plus sham or real tDCS has a transfer effect on C-OCD characteristics. Outcomes will be acquired at pre-, post-, and follow-up assessments. Table 1 shows assessment time points and measures.

**Table 1:**
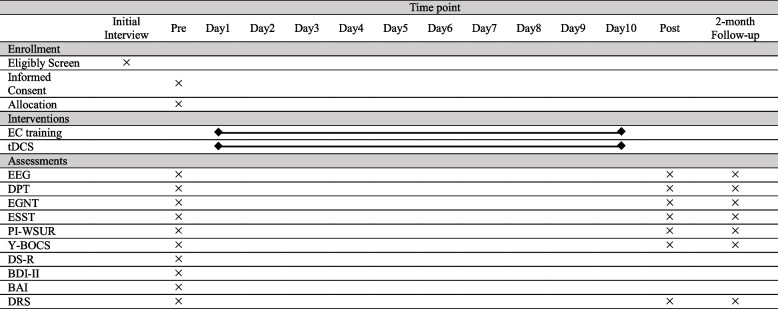
Procedure schedule

*EC*, evaluative conditioning, *tDCS*, transcranial direct current stimulation, *EEG*, electroencephalography, *DPT*, dot-probe test, *EGNT*, Emotional Go/NoGo test, *ESST*, Emotional Stop-Signal test, *PI-WSUR*, Padua Inventory-Washington State University Revision, *Y-BOCS*, Yale-Brown Obsessive-Compulsive Scale, *DS-R*, Disgust Scale-Revised, *BDI-II*, Beck Depression Inventory-II, *BAI*, Beck Anxiety Inventory, *DRS*, Disgust Rating Scale

The primary outcome measures will be disgust reduction and change in clinical symptoms. We hypothesize that pairing C-OCD-related stimuli with positive USs can reduce disgust feeling toward them in C-OCD patients, and simultaneously cathodal tDCS over left OFC may boost this effect. For that, we will measure the amount of disgust elicited by contaminated stimuli on a Disgust Rating Scale, at baseline and after completing interventions. We will also look for possible transfer effects in OCD symptoms from pre- to post-interventions, measured via PI-WSUR and Y-BOCS. Based on previous studies in which the role of disgust has been implied in the pathology of OCD and the assumption that targeting this negative emotion may have treatment outcomes, it is hypothesized that EC may lead to diminishing C-OCD symptoms, mainly when it is along with cathodal tDCS, over left OFC.

The secondary outcomes include cognitive measures and QEEG recording. Disgust as a negative emotion may affect cognitive functions [61, 62], and OCD patient has been shown to have cognitive deficits in attention, memory, and executive function [63–65]. First, we aim to examine attentional bias and inhibitory control in C-OCD patients when confronted with contaminated stimuli compared with neutral ones. Then, the study will explore whether entering these contaminated stimuli into a positive EC training leads to better performance in these cognitive tests. We are also interested in investigating the correlation of cognitive performance level with the self-reported disgust rating, both at the baseline and after completing interventions. For QEEG recording, we will look for changes in brain wave characteristics from pre- to post-interventions.

We expect a more significant effect for all measures when the EC training is combined with cathodal tDCS than each one alone. We will also detect hypothesized effects at a 2-month follow-up.

### Sample size

We calculated the sample size using the G*Power 3.1 software. To be able to get an effect on our primary outcomes (disgust feeling and clinical symptoms) in three measurements (pre-, post-, and follow-up) at repeated-measure-MANOVA approach, with 95% confidence and 80% power, estimating large effect size with Cohen’s *f* value 0.40, a sample size of 46 participants was calculated. With an attrition rate of 20%, we aim to enroll 55 participants.

### Randomization and blinding

After screening for inclusion/exclusion criteria and obtaining informed consent, participants will assign to one of four arms through simple randomization with a 1:1:1:1 ratio using a blocked randomization list created by an online program (sealedenvelope.com) in the blocks of 4. The present study protocol is a single-blind design. The participants will be blind that one or both interventions are sham or active until the end of follow-up assessments. The principal investigator will be responsible for enrolment and assigning participants to interventions.

### Data collection, management, and monitoring

Data from demographic interviews, EEG recordings, cognitive tests, and self-report measures will be collected for all participants. The time points of data collection are shown in Table 1. The participants’ names and contact information will be in an initial form that the principal investigator will keep. Afterward, each participant will be known by a unique numeric identifier, and all data will be stored with that ID. Once a participant completes an assessment session, the data will be transferred to a secure digital database. The database will include EEG records, output files of computerized tests, demographic data that are digitalized manually, and the outputs of self-report measures. The online self-reports will contain a unique ID with no additional personal information. The trial management group (TMG) convenes every week and the trial steering committee (TSC) meets every month to review the implementation of the trial. The TMG is responsible for managing and overseeing the daily operations and activities of the trial. They are responsible for ensuring that the trial is conducted efficiently and effectively. The TSC, consisting of a psychiatrist, a psychologist, a neuroscientist, and a medical engineer, approves TMG actions and provides advice. In addition, they provide reports for the ethics committee and funders. A data monitoring committee (DMC) also meets every six months during the trial, as the trial is considered a low-risk intervention. This committee monitors the completeness of informed consents, data safety, and correspondence with the ethics committee. In case of interim results, the DMC decide to terminate the trial.

### Statistical analysis

First, data entry will be double-checked. Data quality will be examined regarding data distribution, and outliers will be excluded from further analysis. When these items are ensured, the primary and secondary outcomes will be analyzed in detail using IBM SPSS statistics 25 for Windows (Armonk, NY: IBM Corp). Descriptive statistics (e.g., mean and standard deviation) will be reported for each arm separately. We will conduct the analysis within the intention-to-treat (ITT) framework to investigate the effectiveness of the interventions for all subjects with multiple imputed data sets in case of missing data. The appropriate statistical method will be performed to analyze data. The major one will include mixed and repeated measures of multivariate analysis of variance (MANOVA) to assess the effect of group (arm) and time and their interactions on the result of EEG recordings, computerized tests, and self-report measures as dependent variables. The significant level will be set at 0.05. The covariates, including age, disgust sensitivity, anxiety, and depression, will be considered in the statistical model.

### Adherence and retention

At the initial interview, the subjects will be asked about their willingness to participate in the trial and their commitment. They will be explained about the total procedure and the importance of adherence. It will be assured that they understand random allocation and accept that. The time and date of the next session will be discussed at the end of each session, and the participants will receive a reminder the night before. The follow-up appointment will be set through a phone call 1 week before, and the participants will be reminded via a message the night before.

### Patient and public involvement

We collected picture series, especially disgusting ones, according to the input received from C-OCD patients, which established the main basis of the current study. Patients and/or the public did not contribute to the research design, implementation, reporting, or dissemination of the study design. We will collect feedback on patient satisfaction and the intervention effects during the trial.

### Trial status

Recruitment was started in January 2023. The last follow-up data is predicted to be collected by the end of September 2023.

## Discussion

Considering the central role of disgust in developing, maintaining, and treating OCD, this RCT will explore the potential effect of evaluative conditioning training to reduce disgust in C-OCD patients. The proposed EC training program is a computerized task in 4 difficulty levels using contamination-related pictures (as CSs). The pictures will be about those stimuli that most people with C-OCD are concerned about, experience a high degree of disgust when confronted with, and try to prevent. The study will elucidate whether repetitive pairing of disgusting contamination-related stimuli with pleasant ones can modify the disgust experience in this clinical sample.

We aim to try a new intervention method directly targeting disgust in a computerized framework. The novelty of our protocol is administering such a disgust reduction-focused EC training in C-OCD patients and combining it with a brain stimulation protocol that modulates OFC activation.

We also hypothesize that if the disgust experience is successfully modified in this manner, this may decrease obsessions with contamination and compulsive behavior toward them. So, the study will allow for inspecting any decrease in clinical symptoms of C-OCD after the intervention.

Since disgust experience can cause attentional bias and interfere with inhibitory control, this study will provide information about the assumption that EC training focused on disgust reduction may decrease attentional bias toward them and improve inhibitory control. Besides looking for data based on disgust rating through a conscious self-report, cognitive tests will allow us to investigate some implicit aspects. We will also explore the brain wave characteristics to determine whether there is any change due to interventions.

In sum, this trial will investigate the EC effect alone and in combination with cathodal tDCS over OFC on self-report disgust experience, cognitive functions, and clinical symptoms of C-OCD in such a group of patients. Improving any aspect would lead us to make more effective add-on treatments for OCD.

### Supplementary Information

**Additional file 1.**

## Data Availability

Requests of the datasets will be reviewed by the corresponding author. Any available data will be anonymous.

## Abbreviations

BAI: Beck Anxiety Inventory
BDI-II: Beck Depression Inventory-II
CBT: Cognitive behavioral therapy
C-OCD: Contamination-based obsessive-compulsive disorder
CS: Conditioned stimulus
DLPFC: Dorsolateral pre-frontal cortex
DPT: Dot-probe test
DRS: Disgust Rating Scale
DSM-5-TR: The Diagnostic and Statistical Manual of Mental Disorders, Fifth Edition, Text Revision
DS-R: Disgust Scale-Revised
EC: Evaluative conditioning
EEG: Electroencephalography
EGNT: Emotional Go/NoGo Test
ERP: Exposure-response prevention
ESST: Emotional Stop-Signal test
MDD: Major depression disorder
nUS: Neutral valence unconditioned stimulus
OCD: Obsessive-compulsive disorder
OFC: Orbitofrontal cortex
PI-WSUR: Padua Inventory-Washington State University Revision
pUS: Positive valence unconditioned stimulus
qEEG: Quantitative electroencephalography
RCT: Randomized control trial
pre-SMA: Pre-supplementary motor area
tDCS: Transcranial direct current stimulation
tES: Transcranial electric stimulation
USC: Unconditioned stimulus
VR: Virtual reality
Y-BOCS: Yale-Brown Obsessive-Compulsive Scale
